# Genomic surveillance of SARS-CoV-2 tracks early interstate transmission of P.1 lineage and diversification within P.2 clade in Brazil

**DOI:** 10.1371/journal.pntd.0009835

**Published:** 2021-10-13

**Authors:** Alessandra P. Lamarca, Luiz G. P. de Almeida, Ronaldo da Silva Francisco, Lucymara Fassarella Agnez Lima, Kátia Castanho Scortecci, Vinícius Pietta Perez, Otavio J. Brustolini, Eduardo Sérgio Soares Sousa, Danielle Angst Secco, Angela Maria Guimarães Santos, George Rego Albuquerque, Ana Paula Melo Mariano, Bianca Mendes Maciel, Alexandra L. Gerber, Ana Paula de C. Guimarães, Paulo Ricardo Nascimento, Francisco Paulo Freire Neto, Sandra Rocha Gadelha, Luís Cristóvão Porto, Eloiza Helena Campana, Selma Maria Bezerra Jeronimo, Ana Tereza R. Vasconcelos

**Affiliations:** 1 Laboratório de Bioinformática, Laboratório Nacional de Computação Científica, Petrópolis, Brazil; 2 Laboratório de Biologia Molecular e Genômica, Universidade Federal do Rio Grande do Norte, Natal, Brazil; 3 Laboratório de Endemias, Núcleo de Medicina Tropical, Centro de Ciências da Saúde, Universidade Federal da Paraíba, João Pessoa, Brazil; 4 Laboratório de Biologia Molecular, Centro de Ciências Médicas, Universidade Federal da Paraíba, João Pessoa, Brazil; 5 Laboratório de Histocompatibilidade e Criopreservação, Universidade do Estado do Rio de Janeiro, Rio de Janeiro, Brazil; 6 Laboratório de Farmacogenômica e Epidemiologia Molecular, Universidade Estadual de Santa Cruz, Ilhéus, Brazil; 7 Instituto de Medicina Tropical do Rio Grande do Norte, Universidade Federal do Rio Grande do Norte, Natal, Brazil; 8 Departamento de Bioquímica, Centro de Biociências, Universidade Federal do Rio Grande do Norte, Natal, Brazil; University of Tennessee Health Science Center College of Medicine Memphis, UNITED STATES

## Abstract

The sharp increase of COVID-19 cases in late 2020 has made Brazil the new epicenter of the ongoing SARS-CoV-2 pandemic. The novel viral lineages P.1 (Variant of Concern Gamma) and P.2, respectively identified in the Brazilian states of Amazonas and Rio de Janeiro, have been associated with potentially higher transmission rates and antibody neutralization escape. In this study, we performed the whole-genome sequencing of 185 samples isolated from three out of the five Brazilian regions, including Amazonas (North region), Rio Grande do Norte, Paraíba and Bahia (Northeast region), and Rio de Janeiro (Southeast region) in order to monitor the spread of SARS-CoV-2 lineages in Brazil in the first months of 2021. Here, we showed a widespread dispersal of P.1 and P.2 across Brazilian regions and, except for Amazonas, P.2 was the predominant lineage identified in the sampled states. We estimated the origin of P.2 lineage to have happened in February, 2020 and identified that it has differentiated into new clades. Interstate transmission of P.2 was detected since March, but reached its peak in December, 2020 and January, 2021. Transmission of P.1 was also high in December and its origin was inferred to have happened in August 2020. We also confirmed the presence of lineage P.7, recently described in the southernmost region of Brazil, to have spread across the Northeastern states. P.1, P.2 and P.7 are descended from the ancient B.1.1.28 strain, which co-dominated the first phase of the pandemic in Brazil with the B.1.1.33 strain. We also identified the occurrence of a new lineage descending from B.1.1.33 that convergently carries the E484K mutation, N.9. Indeed, the recurrent report of many novel SARS-CoV-2 genetic variants in Brazil could be due to the absence of effective control measures resulting in high SARS-CoV2 transmission rates. Altogether, our findings provided a landscape of the critical state of SARS-CoV-2 across Brazil and confirm the need to sustain continuous sequencing of the SARS-CoV-2 isolates worldwide in order to identify novel variants of interest and monitor for vaccine effectiveness.

## Author summary

Since its first detection in December 2019, the SARS-CoV-2 has evolved into more than a thousand recognized lineages. Several of these lineages are known to have higher transmissibility or better escape from the immune system. One of them is the P.1 lineage, also known as the variant of concern Gamma. It was first discovered in Brazil in December 2020 and has quickly replaced the previous lineage dominating the country, P.2. We used genomic data from SARS-CoV-2 samples that were collected in the first months of 2021 to analyze how P.1, P.2 and other lineages had spread across Brazil. Our research has identified that P.1 lineage was already present in several states of Brazil almost two months before its first detection in the state of Amazonas. Our work sheds light on the importance of continuous monitoring of SARS-CoV-2 lineages in historically understudied regions to early detect and control the spread of new variants of concern.

## Introduction

More than a year after the first case of SARS-CoV-2 infection in Brazil, the country is in a catastrophic situation with 19 million cases of COVID-19 and 550,000 deaths (https://coronavirus.jhu.edu/map.html). The initially dominant lineages B.1.1.28 and B.1.1.33 [1] have been replaced first by the variant P.2 and later by the new variant of concern P.1 (Gamma) [2–4]. P.2 was firstly reported in November 2020 in samples from the state of Rio de Janeiro and was estimated to have first diverged in late July [5]. By December 2020, it was already prevalent in samples from the Brazilian states of Rio Grande do Sul Amazonas and Rio de Janeiro [3,6,7]. P.1 was first detected in the state of Amazonas in mid-December 2020, with a proposed emergence around November [3,4]. Both lineages evolved within the B.1.1.28 clade and convergently carried the E484K mutation in the receptor-binding domain (RBD) of the Spike protein. In addition to E484K, P.1 harbors the N501Y and K417T mutations in the RBD region. It is suggested that those three mutations allow SARS-CoV-2 to better escape from the host’s immune response [8–10]. This hypothesis is supported by the explosive spread of P.1 cases across Brazil and reports of reinfection involving both P.1 and P.2 lineages [11,12].

During the first phase of the COVID-19 pandemic in Brazil, long-distance travel between large urban cities in southeastern states and less populated states from North and Northeast regions played an important role in the explosion of cases across the country [1,13,14]. Since mid-November, there has been a new surge in COVID-19 cases in Brazil, prompting the delimitation of a second phase of the pandemic in the country. This sharp increase in cases is attributed to the emergence of P.1 lineage, which has been reported in several cities in Brazil since December [15–20]. Unfortunately, lineage pervasiveness and genomic diversity are still unknown or outdated in several Brazilian states. If the aforementioned mutations in P.1 and P.2 indeed promote escape from the host’s immune response, this information is crucial to elaborate measures to slow nationwide and worldwide spread.

Monitoring P.1 lineage in Brazil is mainly executed by positive-PCR screening with mutation-targeted primers [18,21–23]. Although this strategy is valuable to estimate the relative frequency of the chosen variant in the screened population, it fails if the primer’s target mutates. Furthermore, the exclusive use of targeted screening prevents monitoring the dispersal and prevalence of other lineages. In this context, sequencing of SARS-CoV-2 genomes systematically sampled from the population is decisive in identifying new variants. In order to reduce the knowledge gap regarding lineage distribution in Brazil during the second surge in COVID-19 cases, we performed an epidemiological and genomic survey by sequencing 185 new SARS-CoV-2 genomes from three Brazilian regions, including states of Amazonas (North region), Rio Grande do Norte, Paraíba, Bahia (all three in the Northeast region) and Rio de Janeiro (Southeast region). Samples were collected between December 2020 and February 2021.

## Materials and methods

### Ethics statement

The present study was approved by Ethical Review Board/Brazilian Commission of Ethical Study (Research Ethics Committee of: Universidade Federal Rio Grande do Norte—CAAE 36287120.2.0000.5537, CAAE 32049320.3.0000.5537, Universidade Federal da Paraíba—CAAE 30658920.4.3004.5183, Universidade Estadual do Rio de Janeiro—CAAE 30135320.0.0000.5259 and Universidade Estadual de Santa Cruz—CAAE 39142720.5.0000.5526). Research protocol was approved without informed consent in accordance with Brazilian National Health Council’s Resolution 510/2016. All samples were residual COVID-19 clinical diagnostic samples de-identified before receipt by the researchers.

### Sample collection

In this work, a total of 185 participants were selected from Amazonas (4), Rio Grande do Norte (44), Paraiba (43), Bahia (58), and Rio de Janeiro (36) states, representing the Brazilian North, Northeast, and Southeast regions. Samples from Amazonas) were obtained from the four patients transferred to Paraíba in late January 2021 while samples from Rio de Janeiro, Rio Grande do Norte, Paraíba and Bahia were randomly selected among COVID-19 positive cases. These samples were collected from December 1st, 2020 through February 15th, 2021. Participants were divided into ninety-two males and 93 females, with age ranging between 11–90 years and with CT values between 8.70 and 29.00 (S1 Table). Nasopharyngeal swabs were obtained from each participant and SARS-CoV-2 infection was diagnosed by RT-PCR using CDC/EUA protocol [24], OneStep/COVID-19 (IBMP, Brazil) Allplex 2019-nCoV (Seegene, South Korea) or nCoVqRT-PCR kits (Biomanguinhos, Fiocruz, Rio de Janeiro).

### Next-generation sequencing and bioinformatics analysis

cDNA synthesis and viral whole-genome amplification were carried out following the Artic Network protocol (https://artic.network/ncov-2019). Amplicon libraries were prepared using the Nextera DNA Flex kit (Illumina, USA). Sequencing was performed in a MiSeq System using MiSeq Reagent Kit v3 (Illumina, USA). Bioinformatic analysis was performed using an in-house pipeline for NGS data pre-processing, variant calling, and genome assembly as previously described [5,6,25]. Briefly, we first inspected the quality control of NGS raw read files in FASTQ format using FastQC (https://www.bioinformatics.babraham.ac.uk/projects/fastqc/) and removed low-quality, bad-formed and optical duplicates in 5’ primer regions sequences with Trimmomatic v0.39 (parameters AVGQUAL = 25 and MINLEN = 100) [26], cutadapt v2.1 and clumpify v38.41 (https://sourceforge.net/projects/bbmap/), respectively. After that, the remaining reads were mapped to the Wuhan-Hu-1 (NC_045512.2) reference genome using BWA v0.7.17 [27]. The BAM files generated in the previous step were sorted and indexed using samtools v.1.11 [28]. We also used GATK v4.1.7.0 to perform the variant calling and filtration [29] and snpEff/SnpSiff v5.0e for VCF annotation. We then combined the list of variants identified in each sample to generate the consensus sequences with bcftools v.1.9 and bedtools v2.29.2 [30–32]. The raw sequencing files and the assembled genomes were submitted to the NCBI and GISAID public databases (NCBI BioProject ID: PRJNA752057, S1 Table)

### Phylogenetic analyses

The evolutionary position of the newly sequenced genomes was inferred using 1441 sequences from Brazil and 70 from other countries, all of them obtained from the GISAID database on February 25th, 2021. The Brazilian background sequences were selected following the strategy described by Paiva et al. [33]. We modified this protocol by clustering aligned sequences with similarity of 0.99985 with CD-hit [34], keeping only the oldest record of each cluster and removing restrictions by country. Global sequences were added by selecting the sequence with the oldest sampling date in GISAID for each lineage found in the Brazilian background dataset. Genome sequence from Wuhan-Hu-1 (NC_045512.2) sample was then added as an outgroup. All sequence alignment steps were conducted using MAFFT with—auto and—addfragments parameters [35]. We used IQ-TREE2 [16] to infer the phylogeny of the final alignment. The substitution model GTR+F+I was selected with ModelFinder [36] using the global sequences as a proxy for the genomic diversity within the larger alignment. Clade support was estimated using 1,000 replicates of bootstrap. To confirm the monophyly of P.7 and N.9 clades, we have also reconstructed their phylogenies with an expanded dataset to include all available sequences in GISAID that share their characteristic mutations. The substitution models for these reconstructions were the GTR+F+I for the P.7 clade and the TIM2+F+I for N.9, both also selected using ModelFinder in IQ-TREE2.

We extracted P.1 and P.2 clades from the complete maximum likelihood phylogeny to infer divergence dates and ancestor spatial dispersion of both lineages with BEAST v1.10.4 [37]. After evaluating with TempEst [38] the correlation between root-to-tip distances and sampling dates (S1 Fig), we selected the strict clock model to date P.1 divergence and the lognormal uncorrelated clock for P.2 [39]. Models used in both analyses were Cauchy’s relaxed random walk for geographic coordinates [40,41], the GTR+F+I substitution model and the exponential growth coalescent tree prior. All models were employed with default parameters. The MCMC was run through 10,000,000 steps with sampling every 10,000th and a 10% burn-in of the posterior results. We extracted ancestor location coordinates using the SERAPHIM package [42] in R software. Vector and raster map data used to plot dispersal routes were obtained from Natural Earth using the R package *rnaturalearth* and can be found on https://naturalearth.s3.amazonaws.com/10m_cultural/ne_10m_admin_1_states_provinces.zip.

To account for the impact of sampling bias across different Brazilian states on the inference of dispersal routes, we have created ten replicates of the previous analyses by resampling the available P.2 (new *n* = 150) and P.1 (50) genomes weighted by the ratio between the number of cases in each state and number of available genomes in GISAID from each state. Because we only used ingroup sequences, the strict clock model was employed on both datasets to infer their divergence dates. Except for this difference, Bayesian analyses were run with the same model and parameters previously described.

## Results

**Fig 1:**
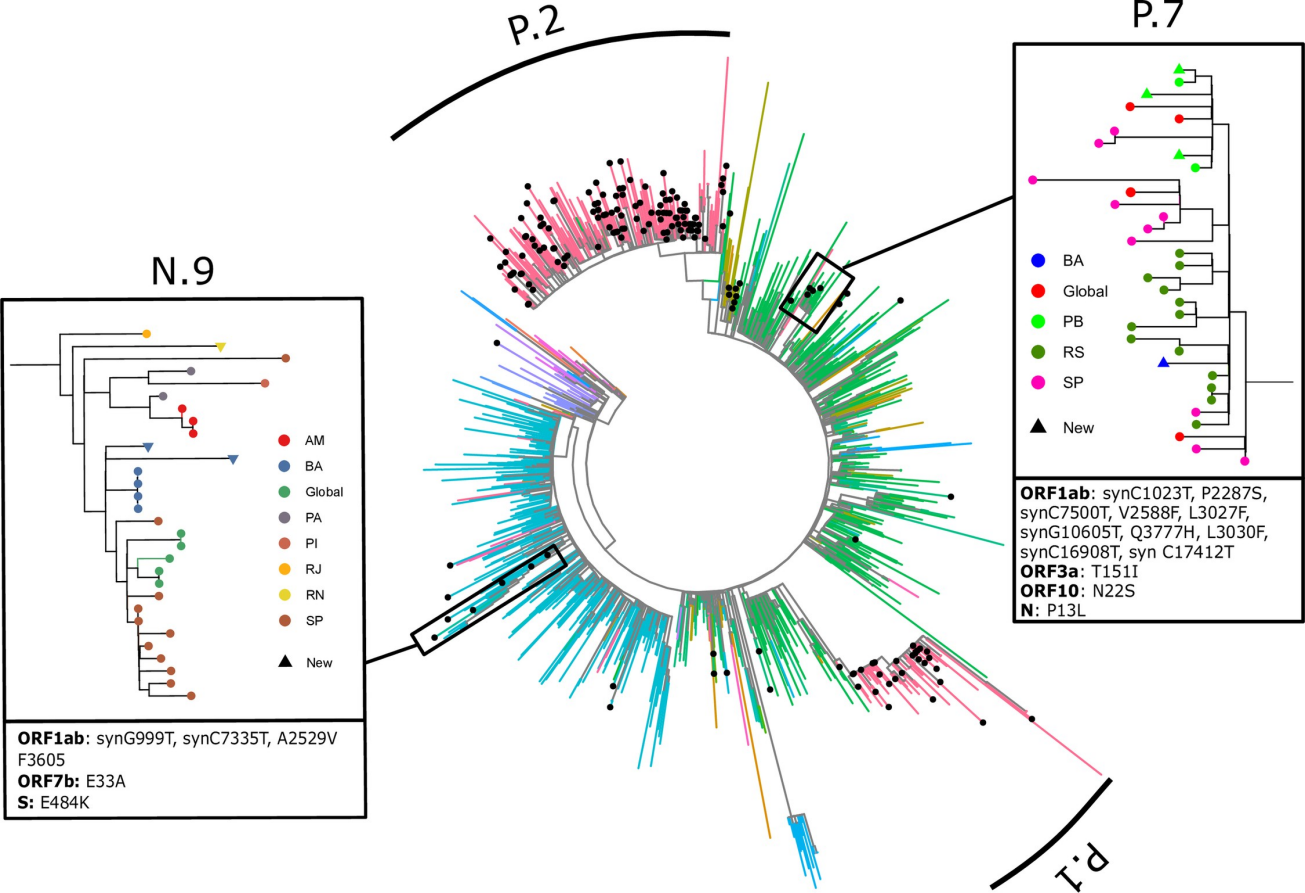
Phylogeny of 1696 SARS-CoV-2 genomes. Newly sequenced samples are signalized by a black point at the tip and lineages P.1 and P.2 are indicated by curved bars. In detail, the N.9 clade, originated from B.1.1.33 (blue branches in the larger tree), and the P.7 clade, originated from B.1.1.28 (green). Colors in smaller trees indicate the Brazilian state in which the sample were collected (AM: Amazonas, BA: Bahia, PA: Pará, PB: Paraíba, PI: Piauí, RJ: Rio de Janeiro, RN: Rio Grande do Norte, RS: Rio Grande do Sul, SP: São Paulo). The boxes below the trees contain the characteristic mutations of each lineage.

The 185 newly sequenced genomes were assigned to 11 different lineages (Figs 1 and S2), with the majority belonging to P.1 (15.68%) and P.2 lineages (64.32%). Other lineages found were B.1.1.143 (4.32%), B.1.1.33 (3.24%), B.1.1.28 (2.70%), B.1.1.29 (2.70%), P.7 (2.16%), B.1 (1.62%), N.9 (1.08%), B.1.1.306 (0.54%), B.1.1.314 (0.54%), B.1.1.34 (0.54%) and B.1.212 (0.54%). The within-state relative lineages frequency revealed that P.2 was the most abundant lineage in Northeast and Southeast regions (S3 Fig). Among the Northeast states, Rio Grande do Norte showed the highest occurrence of the P1 lineage (34.1% of the sequences obtained). Whereas, in the neighboring state of Paraíba, P.2 was the most frequent lineage (51.2% in this study) since late November 2020 [9], and P1 was only reported in early January 2021 (9.3%). Three lineages are described for the first time in the state (B.1.1.29, B.1.1.34 and B.1.212).

We identified 794 single-nucleotide variants (SNVs) across the 185 genomes sequenced, of which 49% were missense substitutions, 45% synonymous and 6% in non-coding regions of the genome (S4 Fig). We found three nonsense mutations in ORF8 (n = 2) and ORF7a (n = 1) in genomes from Rio de Janeiro, Rio Grande do Norte and Paraíba. We observed an elevated accumulation of mutation in the 3’UTR of the genome, mainly targeting ORF3 (subunits a, c and d), ORF9 (b and c), ORF8 and ORF7a (S2 Table). The nucleocapsid (N) protein and the subunit S1 of Spike protein showed the highest accumulation among the structural proteins of the SARS-CoV-2 genome. We found 16 SNVs targeting the receptor-binding domain (RBD) in S1, of which eight were missense variants, including K417T, N439K, L452R, S477R, E484K, N501Y, L518I, A522V.

**Fig 2:**
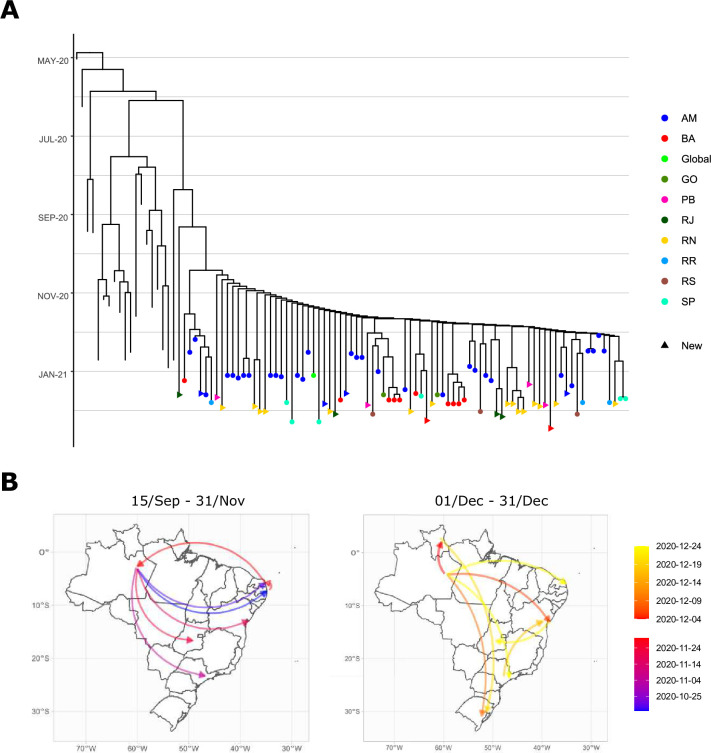
**Divergence times within P.1 lineage (A) and its dispersal routes (B)**. Colors of tip points in the tree indicate the origin of P.1 samples (AM: Amazonas, BA: Bahia, GO: Goiás, PB: Paraíba, RJ: Rio de Janeiro, RN: Rio Grande do Norte, RR: Roraima, RS: Rio Grande do Sul, SP: São Paulo), while tips without a point are sequences from other lineages. Colors of the arrows in the map indicate the date that each interstate transmission route initiated. Vector and raster map data were obtained from Natural Earth and can be found on https://naturalearth.s3.amazonaws.com/10m_cultural/ne_10m_admin_1_states_provinces.zip).

A newly sequenced sample from the state of Rio de Janeiro was recovered as the first divergence within P.1 lineage (Fig 2A). Remarkably, this genome shows traces of intermediary evolution between B.1.1.28 and P.1, harboring 13 out of the 15 lineage-defining mutations according to Pango (https://cov-lineages.org/global_report_P.1.html). We did not observe two mutations (T20N, E92K) characteristic of P.1 clade. The evolutionary position of this new sample was confirmed by repeating the phylogenetic inference analysis with higher P.1 sampling and recovered the same results described here. This newly observed divergence pulls the estimated origin of P.1 lineage to mid-August 2020. Accordingly, interstate dispersal begins in September, with P.1 leaving the state of Amazonas to northeastern states of Rio Grande do Norte and Paraíba (Fig 2B). The divergence between previously sequenced P.1 would happen in mid-October, giving rise to the most common variant. The lineage was already widely distributed across the country by November, with transmission originating in several states, including a reintroduction from Rio Grande do Norte to Amazonas. Interstate transmission reaches its peak in December, with new dispersal routes and maintenance of previous ones. Resampling of the analyzed sequences recovers similar routes to the one obtained with the entire dataset (S5 Fig).

While P.1 sequences are very similar due to their recent origin, we observed a clear evolutive differentiation within P.2 lineage. The first within-clade diversification was estimated to have occurred in late February and the lineage went unreported until December [2], resulting in an uncontrolled transmission across the country (Fig 2B). The first introduction occurs simultaneously in São Paulo and Rio de Janeiro, followed by transmission to Brazil’s southernmost state of Rio Grande do Sul. From then onwards, a multitude of dispersal routes is observed between states. Mirroring P.1 behavior, interstate transmission of P.2 was also most intense during December and extended into January 2021. Once again, the resampling of sequences resulted in similar routes to the ones inferred with the complete dataset (S5 Fig).

We have identified a monophyletic clade of 15 sequences containing the characteristic mutations of lineage N.9, including the E484K mutation. To confirm its monophyly, we have reconstructed this clade’s phylogeny while further increasing the sampling of N.9 sequences to contain all genomes with the E484K available at GISAID (Fig 1). All these extra samples fall within the described clade. Therefore, the monophyly of the group was not disrupted by either the extensive B.1.1.33 outgroup sampling employed on the larger tree (64% bootstrap support) or by increasing the supposed ingroup (86%). Also noteworthy, one of the newly-sequenced samples from Bahia of to this clade is the single B.1.1.306 reported in this work and additionally harbors a previously undescribed N501Y mutation in this lineage. We hypothesized that this new combination of mutations within B.1.1.33 might be due to the Pango misclassification of this sample. We have also confirmed the monophyletic status (99% bootstrap support) of the proposed lineage P.7 [6], which emerged from B.1.1.28 in Brazil’s southernmost state and is now also spread in the Northeast region. This result was, again, recovered even after increasing ingroup sampling with additional sequences available in GISAID. Finally, we report the occurrence of a single sample from the state of Rio Grande do Norte classified as B.1.1.29 that contains both E484K and N429K, uncharacteristic mutations in the lineage.

## Discussion

The ongoing surge of SARS-CoV-2 in Brazil since the end of 2020 has turned the country into the epicenter of a very fast spread of new variants [3,4]. In the present work, we have conducted genomic surveillance of SARS-CoV-2 spread and evolution in historically undersampled regions of Brazil. We have reconstructed past interstate transmission routes across Brazil through phylodynamic analyses of P.1 and P.2 lineages. We have also inferred the origin of P.1, suggested to be the cause of a drastic resurgence in COVID-19 cases [43], to have occurred around August. In contrast, phylogenetic analyses of P.2 indicate that the lineage originated in February 2020, when the virus was first reported in the country and is evolving into differentiated clades.

Our genomic surveillance has evaluated the frequency of lineages currently circulating in each sampled state. As expected, proximity to the Amazonas state seems to be correlated to the pervasiveness of P.1 lineage, as exemplified by the variation observed between Rio Grande do Norte, Paraíba and Rio de Janeiro. The relatively low frequency of P.1 and high frequency of P.2 in our sample from the south of the state of Bahia, a region distant from large airports, may shed light on a much more complex relation between traveling and viral dynamics rather than guilt by association (i.e., mere vicinity). Indeed, previous works suggest that viral spread in smaller or distant cities may happen in a first-come-first-get dynamic, with one lineage overtaking the population [44–46]. Beyond south Bahia cities, this can be seen on Amazonas, where all four samples were from P.1 lineage. Low viral diversity decreases the likelihood of recombination between lineages during a coinfection, which could create new combinations of mutations and more aggressive variants [6]. These results reinforce the importance of local and international traveling restrictions as a preventive measure to slow the spread of the virus [47], measures still not enforced in Brazil and many other countries. As an alternative, policies such as social distancing and early detection of more pathogenic variants could have curtailed the spread of P.1 and P.2 across states and unburdened the public health system [48–50].

**Fig 3:**
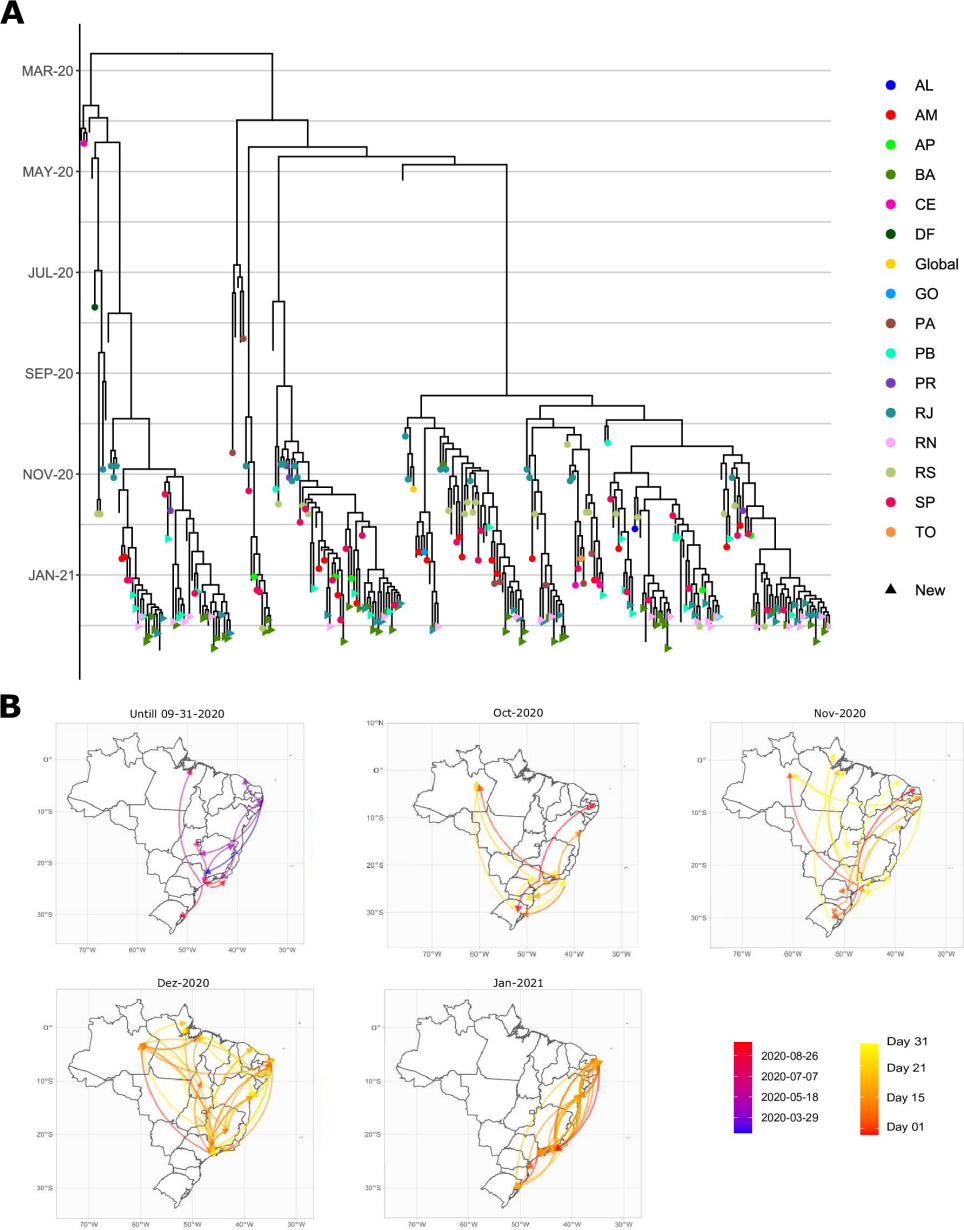
**Divergence times within P.2 lineage (A) and its dispersal routes (B)**. Colors of tip points in the tree indicate the origin of P.2 samples (AL: Alagoas, AM: Amazonas, AP: Amapá, BA: Bahia, CE: Ceará, DF: Distrito Federal, GO: Goiás, PA: Pará, PB: Paraíba, PR: Paraná, RJ: Rio de Janeiro, RN: Rio Grande do Norte, RS: Rio Grande do Sul, SP: São Paulo, TO: Tocantins), while tips without a point are sequences from other lineages. Colors of the arrows in the map indicate the date that each interstate transmission route initiated. Vector and raster map data were obtained from Natural Earth and can be found on https://naturalearth.s3.amazonaws.com/10m_cultural/ne_10m_admin_1_states_provinces.zip.

Some lineages analyzed in this work require attention due to their evolutionary dynamics. First, we observed that P.2 lineage has differentiated in several subclades between April and September of 2020 (Fig 3), all of which are present in many states. The occurrence of P.2 subclades, in practice, means that the expected evolutionary course is for these subclades to evolve into whole new lineages with exclusive mutations. If uncontrolled, epidemiological parameters such as transmission rate, lethality, and immune response escape may vary within the lineage, hindering its containment [51]. Secondly, we have confirmed the spread since December of P.7 [6] to Paraíba, and Bahia’s states, possibly from the Rio Grande do Sul. Not only has it crossed a continental distance, but P.7 has also been detected in England, Japan, and the Netherlands. Higher sampling and investigation of past and present transmission routes is urgent to stop further spread.

The occurrence of lineage N.9, derived from B.1.1.33, in northeastern Brazil has also been demonstrated. According to our evolutionary inference, this lineage may have originated in Rio de Janeiro and disseminated across Brazil. This variant was also detected in samples from the United States, Ireland and Singapore. Remarkably, we observed the convergent occurrence of E484K mutation in this new clade. This mutation was first detected in B.1.351 sequences from South Africa [52] but has now independently emerged in several lineages globally, including P.1 and P.2 [3–5]. Another example of convergent evolution is the single sequence classified as B.1.1.306, which carries not only the mutation E484K inherited from N.9, but also the N501Y variant on the Spike protein gene. N501Y mutation was firstly identified in B.1.1.7 lineage in the United Kingdom [53] and recently detected in the P.1 lineage [3,4]. Finally, the third newly-detected convergent event described in this work is the E484K and N439K variants in a sample of B.1.1.29 from Rio Grande do Norte. The N439K mutation was also first detected at B.1.1.7 samples from the United Kingdom.

Convergent mutations seem to play an essential role in the evolutionary dynamics of SARS-CoV-2. Intense selective pressure from the immune system against prolonged infections may promote intrahost variants with higher adaptive value [25,54–58]. Previous studies have shown that both N501Y and E484K have independently emerged in patients with persistent infection [54,59]. Indeed, all convergent mutations aforementioned are somehow associated with viral escape from immune system response: N439K has shown to escape immune escape from both polyclonal and monoclonal antibodies [60,61]; E484K has been associated with escape from both vaccines and previous infections [2,10,62–64]; and N501Y leads to increased binding specificity to the receptor and is associated with high transmissibility while also escaping immune response [65,66]. Altogether, the combination of these mutations raises the variant’s fitness even higher, and increases the chance of the variant sequence becoming a new and dominant lineage [65]. Continuous monitoring of the convergent sequences here described is fundamental to follow their development and prevent spread in a worst-case-scenario.

Implementing suitable genomic surveillance approaches through sequencing samples selected randomly from PCR-positive tests is a powerful tool to monitor known and new variants across the country. It can guide the elaboration of efficient governmental policies that avoid the collapse of the national healthcare system, as happened in Brazil in the first months of 2021. Both targeted screening and random sampling methods are complementary and congruent to an adequate evaluation of the current pandemic status. Of note, the analyses conducted here are highly dependent on broad sequence sampling through both time and space, which requires both technical and human resources training. Consequently, genomic surveillance is undertaken only by a handful of laboratories, much less than needed to cover a continental-sized country such as Brazil efficiently. This causes a spatial sampling bias, which removes pieces of the historical puzzle that is the reconstruction of dispersal routes. Moreover, the underrepresented states are located in historically underfunded regions, exemplified by the North and Northeastern ones. Scientific collaborations, such as those conducted here, bypass regional barriers to monitor the advances of new and known lineages across states and foment an integrated analysis on the status of the pandemic in the country as a whole. Unfortunately, Brazil has become an open-air laboratory to the emergence and rapid dispersal of novel SARS-CoV-2 variants. Country-wide genomic surveillance is a significant step to better understand the origin and spread of new lineages.

## Supporting information

S1 Fig Correlation between root-to-tip distance and sequence sample dates. Samples P.1 lineage (red) evolved under the same clock dynamics that outgroup sequences (black), whereas P.2 (blue) do not obey the strict clock model.
(TIF)

S2 Fig Evolutionary relationship between the 185 newly-sequenced genomes. Branches of the tree are colored according to the lineage the sequences are classified into.
(TIF)

S3 Fig Frequency of SARS-CoV-2 lineages across Brazilian states. Barplot showing the relative frequency of the 13 lineages found in this study in Amazonas (North region), Rio Grande do Norte, Paraíba, Bahia (all three in the Northeast region), and Rio de Janeiro (Southeast region).
(TIF)

S4 Fig Genomic characterization of SARS-CoV-2 mutations identified. Distribution of single-nucleotide variants (SNVs) found in the 185 genomes sequenced in this study. Each vertical line represents the relative variant frequency in the total number of genomes sequenced and its target protein products. The receptor-binding domain (RBD) highlighted in red showed the main mutations associated with P.2 and the variant of concern P.1. Density plot shows the accumulation of mutations across the SARS-CoV-2 genome.
(TIF)

S5 Fig Dispersal routes of P.1 lineage inferred for the ten subsampled datasets. Colors of the arrows in the map indicate the date that each interstate transmission route initiated. Vector and raster map data were obtained from Natural Earth and can be found on https://naturalearth.s3.amazonaws.com/10m_cultural/ne_10m_admin_1_states_provinces.zip).
(TIF)

S6 Fig Dispersal routes of P.2 lineage inferred for the ten subsampled datasets. Colors of the arrows in the map indicate the date that each interstate transmission route initiated. Vector and raster map data were obtained from Natural Earth and can be found on https://naturalearth.s3.amazonaws.com/10m_cultural/ne_10m_admin_1_states_provinces.zip.
(TIF)

S1 Table Sample information. (XLSX)

S2 Table Frequency of mutations in SARS-CoV-2 genome. (XLSX)

S3 Table Acknowledgement for GISAID samples. (PDF)

S1 File Phylogenetic trees in Figs 1–3, written in NEXUS format. (TXT)
