# Supplementary figures and images for: Genomic surveillance of SARS-CoV-2 tracks early interstate transmission of P.1 lineage and diversification within P.2 clade in Brazil
Source: PLoS Negl Trop Dis. 2021 Oct 13;15(10):e0009835. doi: 10.1371/journal.pntd.0009835 (PMC8544873; doi:10.1371/journal.pntd.0009835)

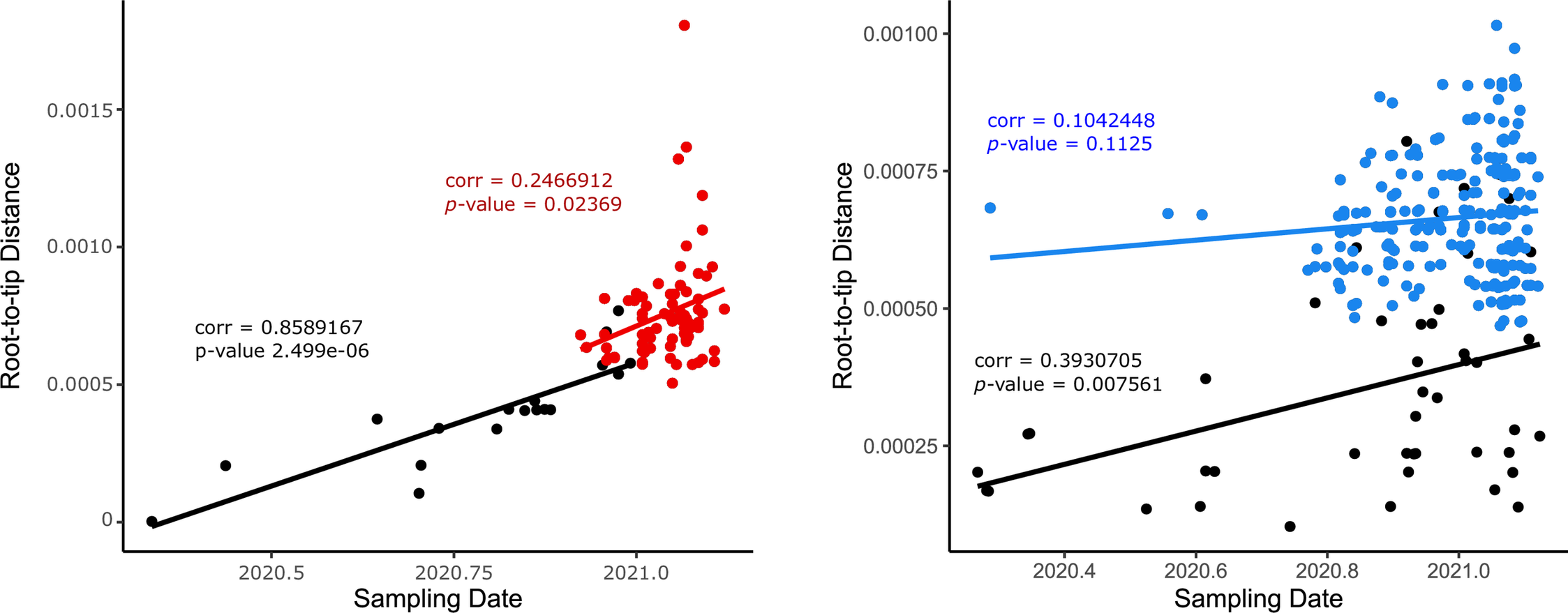

Supplement: S1 Fig — Samples P.1 lineage (red) evolved under the same clock dynamics that outgroup sequences (black), whereas P.2 (blue) do not obey the strict clock model. (TIF) [file pntd.0009835.s001.tif]

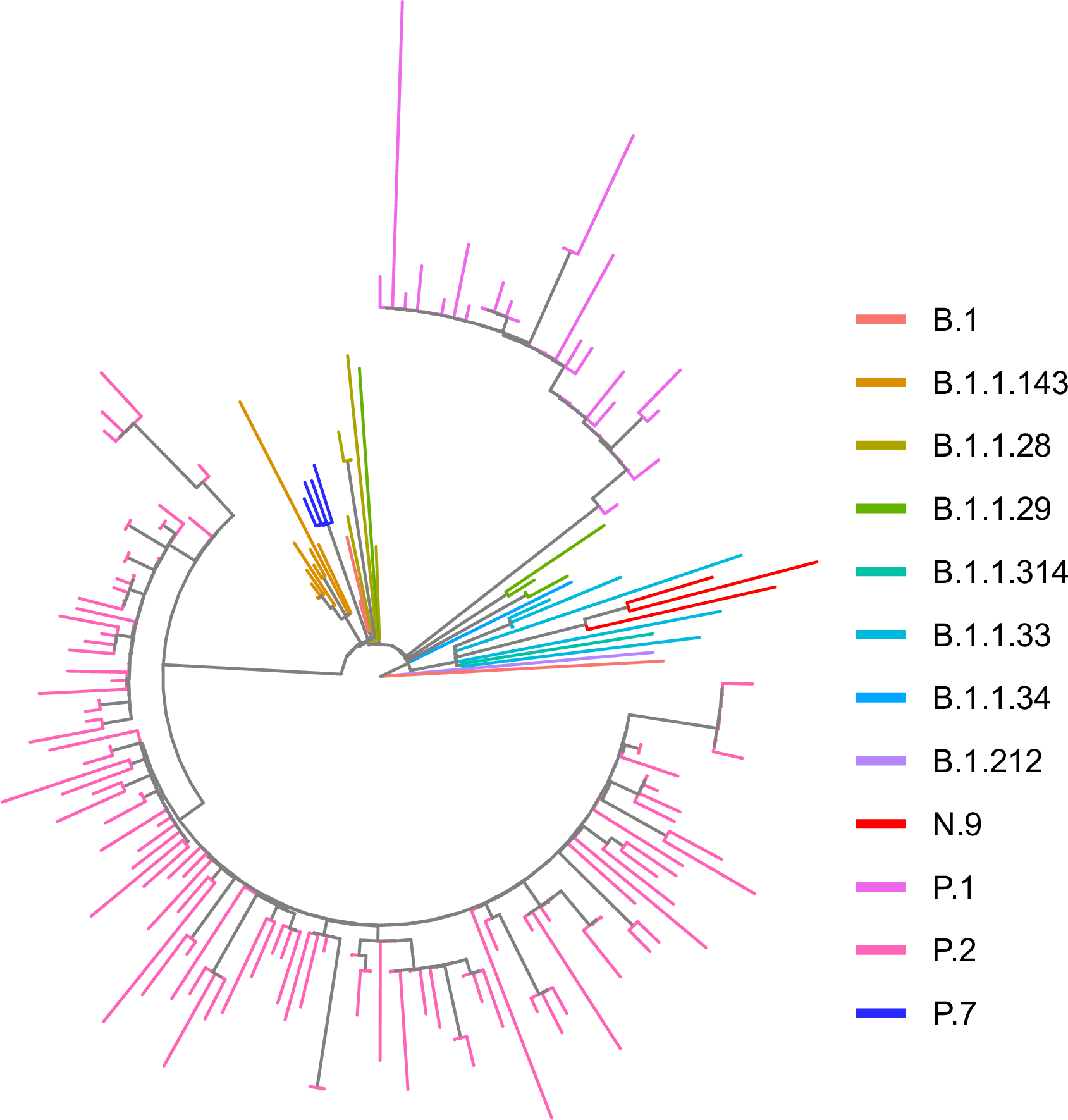

Supplement: S2 Fig — Branches of the tree are colored according to the lineage the sequences are classified into. (TIF) [file pntd.0009835.s002.tif]

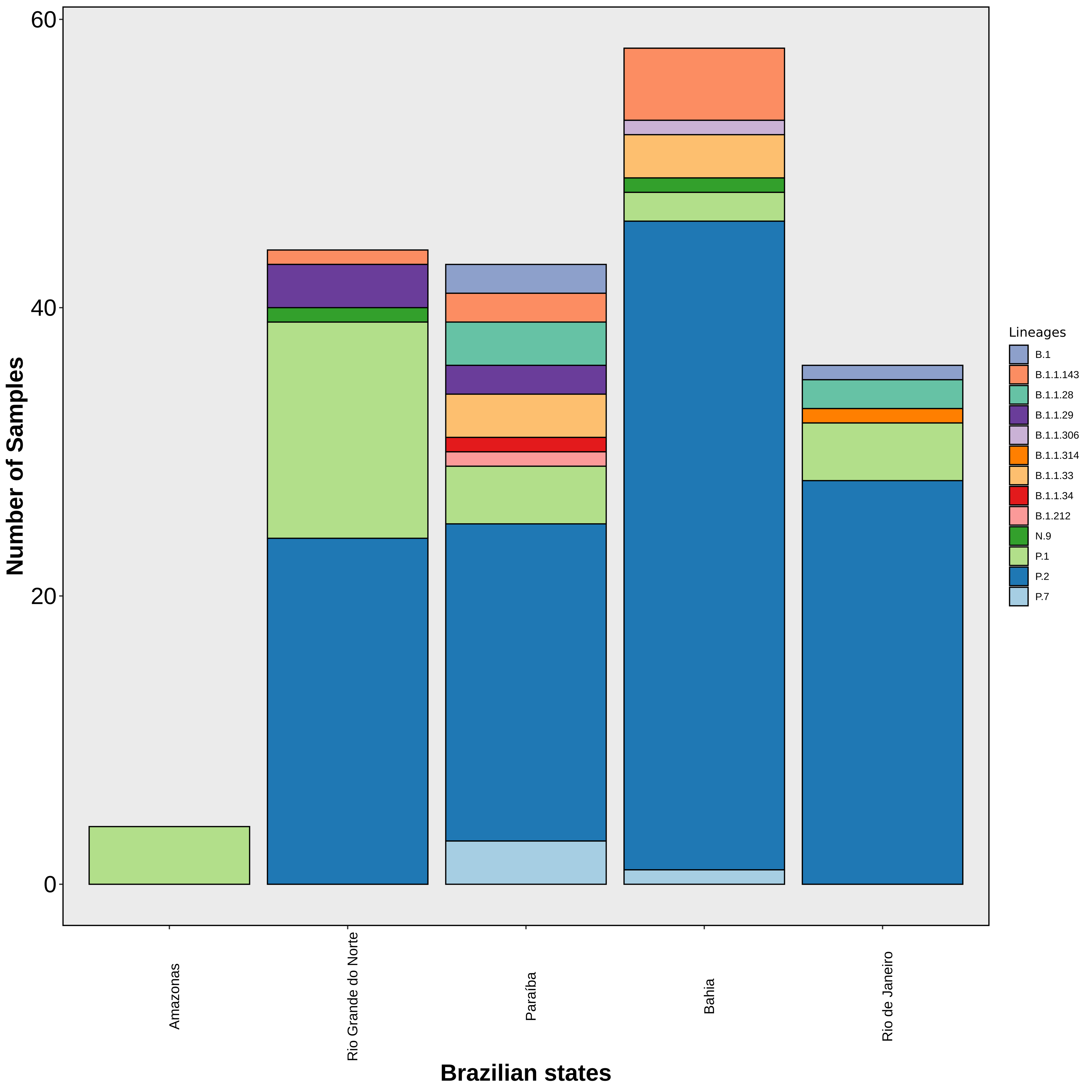

Supplement: S3 Fig — Barplot showing the relative frequency of the 13 lineages found in this study in Amazonas (North region), Rio Grande do Norte, Paraíba, Bahia (all three in the Northeast region), and Rio de Janeiro (Southeast region). (TIF) [file pntd.0009835.s003.tif]

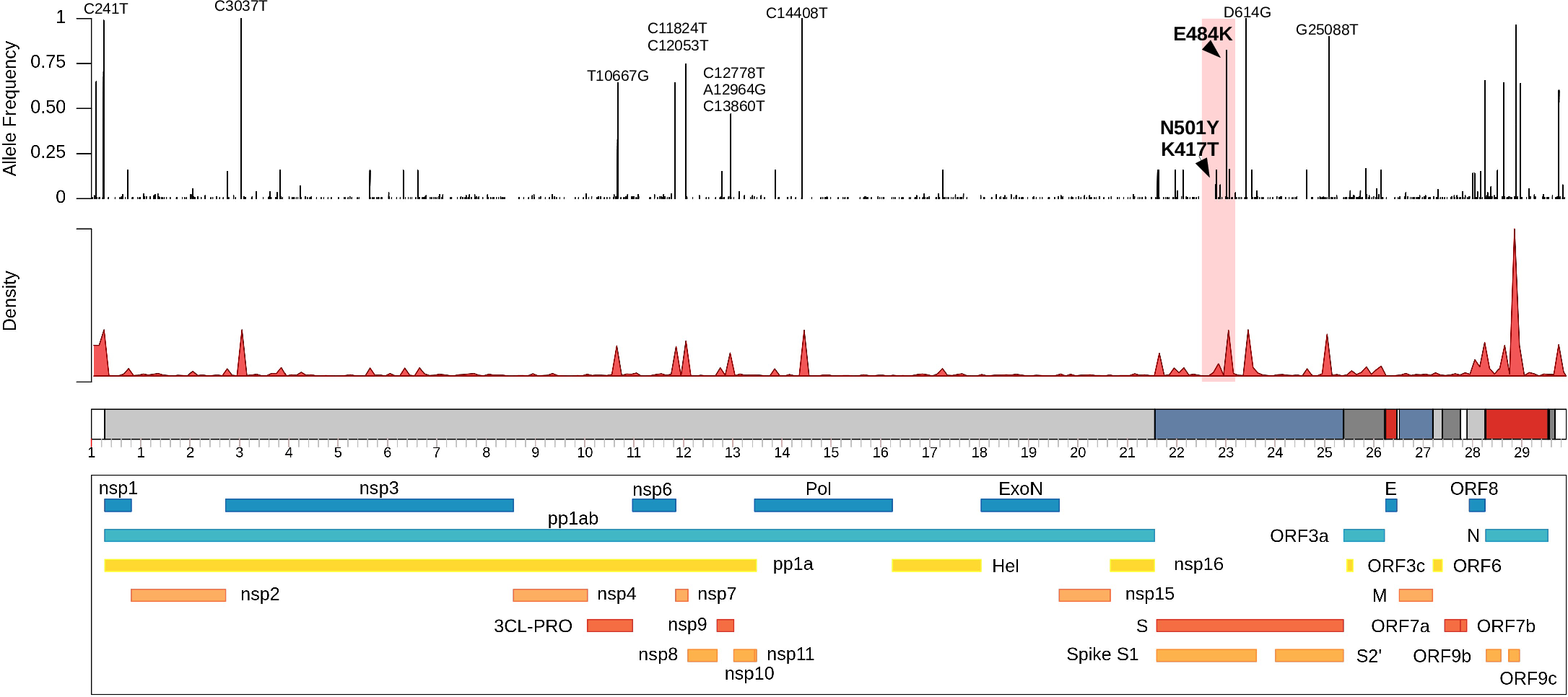

Supplement: S4 Fig — Distribution of single-nucleotide variants (SNVs) found in the 185 genomes sequenced in this study. Each vertical line represents the relative variant frequency in the total number of genomes sequenced and its target protein products. The receptor-binding domain (RBD) highlighted in red showed the main mutations associated with P.2 and the variant of concern P.1. Density plot shows the accumulation of mutations across the SARS-CoV-2 genome. (TIF) [file pntd.0009835.s004.tif]

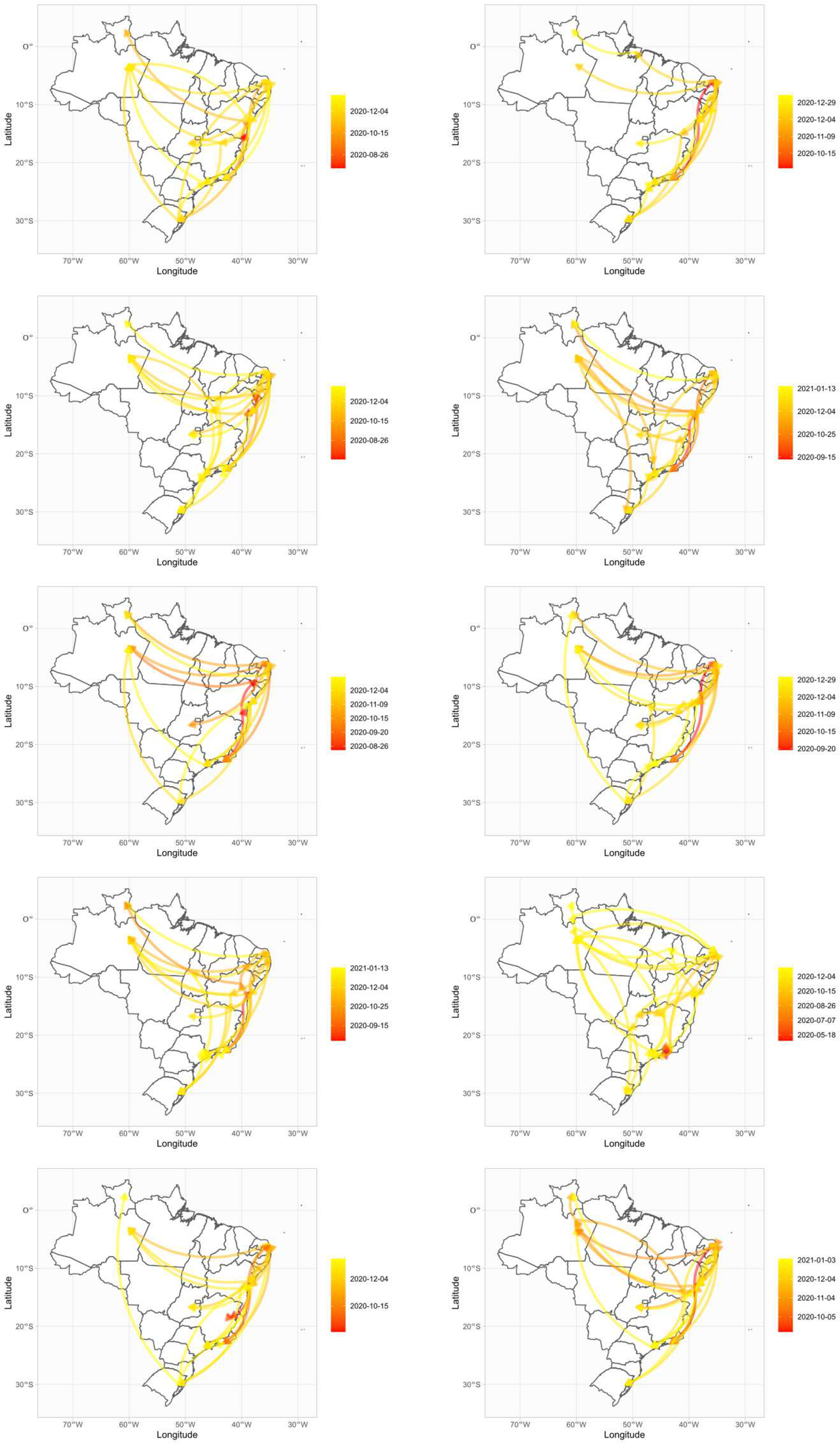

Supplement: S5 Fig — Colors of the arrows in the map indicate the date that each interstate transmission route initiated. Vector and raster map data were obtained from Natural Earth and can be found on https://naturalearth.s3.amazonaws.com/10m_cultural/ne_10m_admin_1_states_provinces.zip). (TIF) [file pntd.0009835.s005.tif]

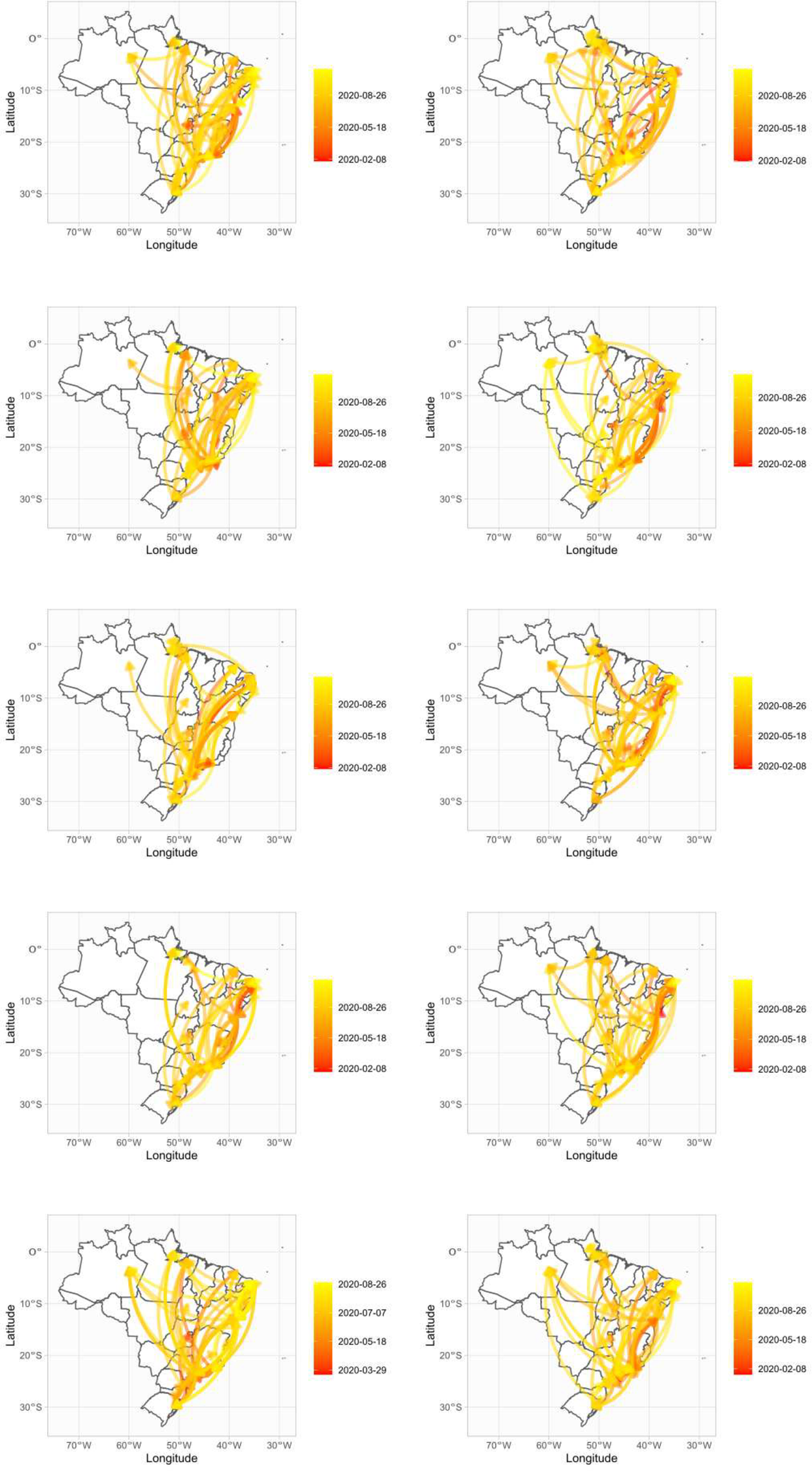

Supplement: S6 Fig — Colors of the arrows in the map indicate the date that each interstate transmission route initiated. Vector and raster map data were obtained from Natural Earth and can be found on https://naturalearth.s3.amazonaws.com/10m_cultural/ne_10m_admin_1_states_provinces.zip. (TIF) [file pntd.0009835.s006.tif]
